# Flexible Thermoelectric Generator Based on Polycrystalline SiGe Thin Films

**DOI:** 10.3390/ma15020608

**Published:** 2022-01-14

**Authors:** Tomoki Ozawa, Masayuki Murata, Takashi Suemasu, Kaoru Toko

**Affiliations:** 1 Institute of Applied Physics, University of Tsukuba, 1-1-1 Tennodai, Tsukuba 305-8573, Japan; ozawa.1997h9@gmail.com (T.O.); suemasu@bk.tsukuba.ac.jp (T.S.); 2 Research Institute for Energy Conservation, AIST, Tsukuba 305-8569, Japan

**Keywords:** polycrystalline SiGe, thin film, layer exchange, low-temperature synthesis, thermoelectric generator, flexible

## Abstract

Flexible and reliable thermoelectric generators (TEGs) will be essential for future energy harvesting sensors. In this study, we synthesized p- and n-type SiGe layers on a high heat-resistant polyimide film using metal-induced layer exchange (LE) and demonstrated TEG operation. Despite the low process temperature (<500 °C), the polycrystalline SiGe layers showed high power factors of 560 µW m^−1^ K^−2^ for p-type Si_0.4_Ge_0.6_ and 390 µW m^−1^ K^−2^ for n-type Si_0.85_Ge_0.15_, owing to self-organized doping in LE. Furthermore, the power factors indicated stable behavior with changing measurement temperature, an advantage of SiGe as an inorganic material. An in-plane π-type TEG based on these SiGe layers showed an output power of 0.45 µW cm^−2^ at near room temperature for a 30 K temperature gradient. This achievement will enable the development of environmentally friendly and highly reliable flexible TEGs for operating micro-energy devices in the future Internet of Things.

## 1. Introduction

Energy harvesting technologies will be essential for creating a sustainable society [1,2,3,4]. In particular, flexible thermoelectric generators (TEGs) will be a key technology for use in ubiquitous sensors and wearable devices in the Internet of Things (IoT) [5]. Direct synthesis of thermoelectric materials on plastic substrates is the most promising approach for fabricating highly versatile flexible TEGs [6]. However, as plastics typically have low heat resistance, active research is ongoing into the low-temperature synthesis of various materials, including organic materials [7], metal compounds [8,9,10], oxides [11], and graphene [12]. Although some candidate materials have demonstrated excellent thermoelectric performance, most of the applications of flexible TEGs, including healthcare, require highly reliable and non-toxic materials.

Inorganic semiconductors have a reputation for environmental resistance and stability. Although III–V compound semiconductors are useful for TEGs [13,14], group IV materials are suitable for electronic devices in close proximity to humans because they are nontoxic and relatively abundant. Moreover, SiGe alloys are the most reliable of the thermoelectric materials, as shown by their decades-long use in space applications [15,16,17]. Thermoelectric SiGe thin films have been formed on insulators using laser sintering [18], sputtering [19,20], chemical vapor deposition [21,22,23,24], electrophoretic deposition [25], solid-phase crystallization [26,27,28], and metal-induced crystallization [29]. However, the formation of crystalline SiGe typically requires high temperatures (>500 °C), while lower temperature processes make it more difficult to activate the dopants in SiGe, thereby reducing the thermoelectric performance. Thus, it has been practically difficult to obtain thermoelectric SiGe films on a plastic substrate, despite SiGe being a promising candidate material for human-friendly wearable TEGs.

However, metal-induced layer exchange (LE) can be used to overcome these issues. When metal and amorphous semiconductor layers are stacked and heated, LE occurs via the following stages: diffusion of semiconductor atoms into the metal layer; generation of semiconductor crystal nuclei in the metal; lateral growth of semiconductor crystals; and extrusion to the top of the metal [30]. Through this reaction between the metal and semiconductor, LE enables low-temperature synthesis and high-concentration doping in polycrystalline SiGe thin films. LE has been used to produce thermoelectric SiGe films using Al [31,32] and Zn [33,34] for p-type, and Zn:As [34] and Ag:As [35] for n-type alloys. Thermoelectric power factors (*PF*s) for layers formed on a glass substrate at 400–500 °C have been measured as 850 μW m^−1^K^−2^ for p-type Si_0.4_Ge_0.6_ [36] and 1000 μW m^−1^K^−2^ for n-type Si_0.85_Ge_0.15_ [35]. These *PF* values were the highest obtained for SiGe thin films formed at temperatures below 1000 °C; however, the synthesis temperatures still exceeded the maximum heat resistance of most plastic substrates.

In this paper, we investigate using LE for the formation of p- and n-type SiGe layers onto a recently developed polyimide film (XENOMAX^®^) with a heat resistance till 500 °C. The resulting flexible TEG based on the SiGe layers demonstrates stable operation with an output power close to 1 μW at approximately room temperature (RT). 

## 2. Materials and Methods

**Figure 1:**
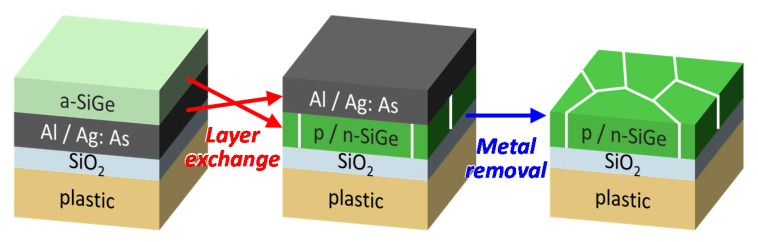
Schematic diagram of the LE process. The metal layer is Al for p-type Si_0.4_Ge_0.6_ and Ag:As for n-type Si_0.85_Ge_0.15_.

Figure 1 presents a schematic of the metal-induced LE process. The p-type and n-type films require equally low resistances to obtain high output power in TEGs and, as resistance is inversely proportional to film thickness, this means thicker films are preferable. Although Al-induced LE can produce SiGe layers as thick as 1000 nm [36], Ag-induced LE is limited to 70 nm under the chosen experimental growth conditions. Therefore, owing to the characteristics of SiGe layers formed on glass [35,36], film thicknesses of 500 nm and 70 nm were chosen, respectively, to obtain approximately equal resistance values for the p-type and n-type films. For p-type, the Al and amorphous (a-) Si_0.4_Ge_0.6_ layers (500 nm thick each) were sequentially prepared at RT without breaking vacuum onto a 38-μm-thick XENOMAX^®^ plastic substrate (Xenomax-Japan Co., Ltd., Fukui, Japan, heat-resistant temperature: 500 °C, thermal expansion coefficient: 3.0 × 10^−6^ K^−1^) covered with 100 nm thick SiO_2_. This was achieved using radio frequency (RF) magnetron sputtering (base pressure 3.0 × 10^−4^ Pa) with Ar plasma. The RF power was set to 50 W for Al and a-Si_1-*x*_Ge*_x_* and 30 W for Ag:As. For n-type, 10% As-doped Ag (Ag:As) and a-Si_0.85_Ge_0.15_ layers (70-nm-thick each) were sequentially prepared under the same conditions. The purity of each material was 99.9%. The samples were then annealed in an N_2_ ambient furnace at 425 °C for 15 h for p-type and 500 °C for 5 h for n-type. These annealing conditions were effective in obtaining a high *PF* for each sample [35,36]. Then, the Ag and Al layers were etched away using an acid solution (H_3_PO_4_:HNO_3_:CH_3_COOH:H_2_O = 16:1:1:2) and HF solution (HF 1.5%), respectively. 

The SiGe layers were evaluated using Raman spectroscopy (wavelength 532 nm and spot size 5 µm) and scanning electron microscopy (SEM, Hitachi High-Tech Corp., Tokyo, Japan) equipped with an electron backscattering diffraction (EBSD) analysis attachment. To evaluate the carrier concentration, the Van der Pauw method was used to carry out Hall effect measurements using the HL5500PC system (Bio-Rad, Hercules, CA, USA) with a 0.32 T permanent magnet. Then, the electrical conductivity *σ* and the Seebeck coefficient *S* were measured using the ZEM-3 system (Advance-Riko, Inc., Yokohama, Japan), where Ag paste was used to fix the sample to a ceramic stage.

Using the p- and n-type SiGe layers, an in-plane π-type TEG with a p-n-p series structure was fabricated. The catalyst metal and SiGe layers were sequentially patterned onto a substrate using metal mask evaporation, and annealed at 425 °C for 15 h followed by 500 °C for 5 h in an N_2_ ambient furnace to induce LE for the p- and n-type SiGe layers. After removing the metal layers using the etching solutions, Ag electrodes (1 µm thick) were prepared using the same sputtering system with a metal mask. The resulting area used for power generation comprised two p-type layers and one n-type layer of 2 × 3 mm^2^ each. The output power was evaluated using a current–voltage measuring device while an indirect resistance heater and Stirling refrigerator (Koyo Thermo Systems Co., Ltd., Nara, Japan) created an in-plane thermal gradient between the heating and cooling regions. A differential thermocouple was mechanically contacted at the sample surface to monitor the temperature. A variable resistance load was connected across the TEG using Cu wires to measure its current–voltage characteristic and output power.

## 3. Results

**Figure 2:**
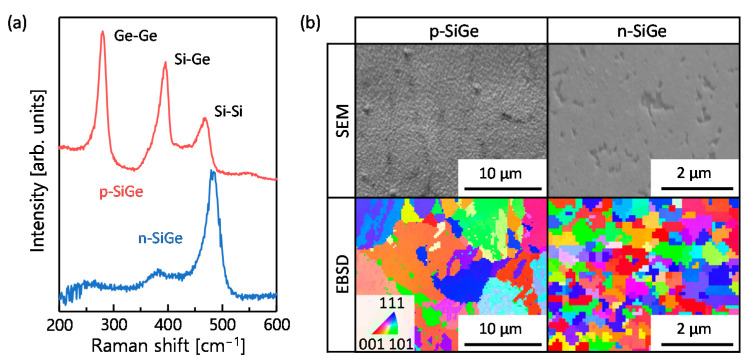
Characteristics of the p- and n-type samples formed by LE using Al and Ag:As, where metal layers were removed. (**a**) Raman spectra and (**b**) SEM (70° tilted) and inverse pole figure images, where colors indicate crystal orientation as shown by color key.

Hall effect measurements confirmed that p-type and n-type SiGe layers were formed through the LE process using Al and Ag:As, respectively, due to Al acting as an acceptor and As acting as a donor for SiGe [35,36]. The carrier mobility, Hall coefficient, and carrier concentration were found to be 19 cm^2^ V^−1^s^−1^, 0.066 cm^3^ C^−1^, and 9.5 × 10^19^ cm^−3^ for the p-type sample, and 4.4 cm^2^ V^−1^s^−1^, 0.23 cm^3^ C^−1^, and 2.7 × 10^20^ cm^−3^ for the n-type sample, respectively. These high carrier concentrations, despite the low process temperature, are attributed to the property that impurities diffuse and activate at the solid solubility limit during LE in SiGe [30]. Figure 2a shows the Raman spectra obtained for the p- and n-type samples after metal removal. Peaks corresponding to Si-Si, Si-Ge, and Ge-Ge vibration modes indicate LE successfully formed crystalline SiGe layers onto substrates for both samples. The SiGe composition, estimated from the Raman spectra [37,38], was almost the same as the as-prepared a-SiGe layers determined by Rutherford backscattering spectrometry. 

The SEM images in Figure 2b show that although the SiGe layers contained some voids, they were generally uniform. No cracking was observed in the SiGe layer as the plastic substrate and Si_1-*x*_Ge*_x_* (4.2–5.8 × 10^−6^ K^−1^, depending on *x*) have similar thermal expansion coefficients. The inverse pole figure images in Figure 2b show that both SiGe layers are polycrystalline with random orientations, with an average grain size of a few micrometers for p-type SiGe and a few hundred nanometers for n-type SiGe. The strong dependence of the grain size on metal type is attributed to the diffusion rates and growth energies of SiGe differing between metals [30]. The grain sizes were slightly smaller than those synthesized on a quartz glass substrate [35,36]. This behavior is a common trend for LE using a plastic substrate [31,33,39], and is likely due to either the surface roughness of the substrate or warpage and shrinkage during heat treatment. Indeed, slight warping was observed for the plastic substrate samples, which increased the difficulty of obtaining accurate thermal conductivity values. For the samples with the quartz glass substrate, the thermal conductivity was 2.2 Wm^−1^ K^−1^ for p-type Si_0.4_Ge_0.6_ [36] and 1.6 Wm^−1^ K^−1^ for n-type Si_0.85_Ge_0.15_ [35], whereas the samples with plastic substrate had lower thermal conductivities than these due to the smaller grain sizes. 

**Figure 3:**
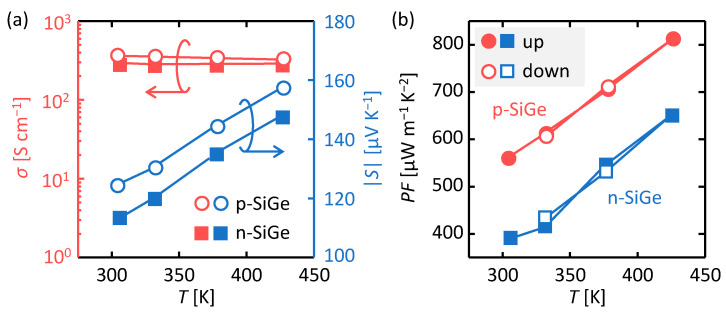
Thermoelectric properties of the p- and n-type SiGe samples formed by LE using Al and Ag:As as a function of measurement temperature (*T*). (**a**) Electrical conductivity *σ* and Seebeck coefficient *S* and (**b**) power factor (*PF*). The data points are averaged values of three measurements, and the error is within acceptable limits.

Figure 3a shows that *σ* is slightly higher for p-type SiGe than for n-type SiGe, despite n-type SiGe having a higher carrier concentration. This is because p-type SiGe has a higher carrier mobility, which likely can be attributed to several features including higher Ge concentration [40], larger grain size (see Figure 2b), thicker film (hence less interfacial carrier scattering) [41], and lower grain boundary potential than for n-type SiGe [42,43]. For both samples, the *σ* values are slightly lower than those of the quartz glass substrate samples [35,36], which is consistent with the grain size trend shown in Figure 2b. Figure 3a also shows the Seebeck coefficient corresponding to the appropriate conduction type obtained for p- and n-type SiGe. Reflecting the carrier concentration, p-type SiGe exhibits a higher |*S*| than n-type SiGe. For increasing measurement temperature (*T*), *σ* decreases whereas |*S*| increases for both p- and n-type SiGe. This is a typical trend for degenerated semiconductors exhibiting metallic behavior [15,16], which is attributed to the high carrier concentration. Figure 3b shows that the power factor at RT is 560 µW m^−1^ K^−2^ for p-type SiGe and 390 µWm^−1^ K^−2^ for n-type SiGe. For both samples, the *PF* was measured while raising *T* from RT to 150 °C and then lowering back to 50 °C. Figure 3b shows the *PF* values increase with increasing *T* due to the increase in *S*, and that both samples continue to exhibit similar *PF* values at each *T* after heating to 150 °C. This result guarantees stable device operation in the expected temperature range of IoT device operation.

**Figure 4:**
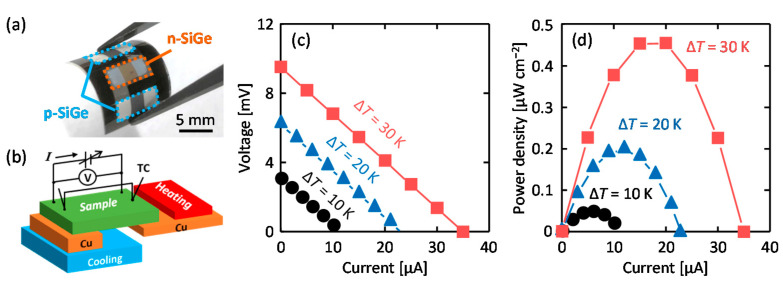
(**a**) Photograph of the flexible TEG using p- and n-type SiGe on a plastic substrate formed by LE using Al and Ag:As. (**b**) Schematic of a system for the output power measurement. (**c**) Voltage–current lines and (**d**) power density–current curves of the TEG obtained at constant temperature differences Δ*T* of 10 K (circles), 20 K (triangles), and 30 K (squares), where the sample stage was kept at 300 K. The data points represent the average of 50 measurements, and the error is within acceptable limits.

Figure 4a shows that the TEG sample maintained flexibility after the device fabrication process. According to our previous study [31], SiGe thin films prepared using the Al-induced LE did not show any performance degradation on bending (up to 120°). Figure 4b shows the setup used to measure the output power. A heater and heat sink were provided on either side to induce a temperature gradient (Δ*T* = 10, 20, and 30 K) across the sample surface, with the sample stage at a constant 300 K. A space was maintained between the copper plate on the heater side and the heat sink to avoid contact. Figure 4c shows that the voltage linearly decreases with increasing current when load resistance is changed. The open-circuit voltage and short-circuit current are almost consistent with the estimated values from |*S*| and *σ* for the SiGe layers (see Figure 3a). As shown in Figure 4d, the power density was derived from the product of the current and voltage values and the area of the SiGe layers, and ideal power-current curves obtained, confirming TEG operation. Increasing the open-circuit voltage resulted in the output power increasing with Δ*T*. The maximum output power of the flexible TEG reached 0.45 µW cm^−2^ at Δ*T* = 30 K, which is close to the power required to operate micro-energy devices such as sensors. This output power far exceeds that of the TEGs previously fabricated on quartz glass substrates using p- and n-type SiGe layers formed by Ag-induced LE [35]. This is attributed to the use of Al-induced LE for the formation of the p-type SiGe layer, which improves *σ* and, therefore, current values for the TEG. 

The power density obtained in this study is comparable to or even better than other energy harvesting technologies such as piezoelectric materials, biofuel cells, solar energy harvesters, and RF harvesters [1,2,3,4]. However, issues remain for practical implementation, in particular ensuring a temperature gradient in the real environment. It is hoped that the evolution of applied research on TEGs will enable the future use of the technology fabricated in this study.

## 4. Conclusions

The p-type and n-type SiGe layers were synthesized on a highly heat-resistant polyimide film using LE and used to fabricate a flexible TEG. Despite a process temperature below 500 °C, the SiGe layers showed high *PF*s of 560 µW m^−1^ K^−2^ for p-type and 390 µW m^−1^ K^−2^ for n-type due to self-organized doping during LE. Furthermore, the *PF*s showed stable behavior with respect to measurement temperature, which is an advantage of SiGe as an inorganic material. The flexible TEG based on the SiGe layers produced an output power of 0.45 µW cm^−2^ at RT for Δ*T* = 30 K. These results will enable the development of environmentally friendly and highly reliable flexible TEGs to operate micro-energy devices in the IoT.

## Data Availability

The data presented in this study are available on request from the corresponding author.
